# Overweight/obesity and dental caries in Brazilian children and adolescents: a systematic review and meta-analysis

**DOI:** 10.1590/1807-3107bor-2024.vol38.0015

**Published:** 2024-03-11

**Authors:** Julia Faria PIZZI, Camila Faria CARRADA, Maria Vitória de Sá ZEFERINO, Paulo Victor Teixeira DORIGUÊTTO, Lucas Guimarães ABREU, Flávia Almeida Ribeiro SCALIONI, Karina Lopes DEVITO, Rosangela Almeida RIBEIRO

**Affiliations:** (a) Universidade Federal de Juiz de Fora – UFJF, School of Dentistry, Juiz de Fora, MG, Brazil.; (b) Faculdade de Ciências Médicas e da Saúde – Suprema, School of Dentistry, Department of Paediatric Dentistry, Juiz de Fora, MG, Brazil.; (c) Centro Universitário Governador Ozanam Coelho – Unifagoc, School of Dentistry; Ubá, MG, Brazil.; (d) Universidade Federal de Minas Gerais – UFMG, Schoool of Dentistry, Department of Oral Health for Children and Adolescents, Belo Horizonte, MG, Brazil.; (e) Universidade Federal de Juiz de Fora – UFJF, School of Dentistry, Department of Social and Paediatric Dentistry; Juiz de Fora, MG, Brazil.; (f) Universidade Federal de Juiz de Fora – UFJF, School of Dentristry, Department of Dental Clinic, Juiz de Fora, MG, Brazil.

**Keywords:** Obesity, Overweight, Dental Caries, Child, Systematic Review

## Abstract

This review aimed to assess the association between overweight/obesity and dental caries in Brazilian children/adolescents. Searches were performed in the Web of Science, Scopus, Cochrane, PubMed, Embase and SciELO, Lilacs and Open Grey literature databases up to June 2022. The Joanna Briggs Institute checklist for analytical cross-sectional studies, the checklist for cohort studies, and the checklist for case-control studies were used. A total of 41 publications were included, and 15 meta-analyses were performed. The authors analyzed the differences in weighted mean difference (MD) and odds ratios (OR), and their corresponding confidence intervals (CI) (95%) for dental caries among eutrophic and obese and/or overweight children/adolescents. Meta-analyses showed that there was no association between overweight and/or obesity and dental caries in Brazilian children/adolescents for most anthropometric reference curves using BMI (Body Mass Index). A greater experience of dental caries was associated with well-nourished adolescents in permanent dentition, compared with obese individuals in the same dentition, as classified by the CDC 2000 curve (OR = 2.53, 95% CI;1.49–4.29; p = 0.0006; I^2^ = 0%) in dichotomous outcome studies, and (MD = 0.61, 95%CI: 0.08–1.15; p = 0.02; I^2^ = 0%) in continuous studies. The strength of the evidence of the results was classified as very low, low or moderate. It was concluded that there is no association between overweight and/or obesity and dental caries in Brazilian children/adolescents for most anthropometric reference curves using BMI. A greater experience of dental caries was associated with well-nourished adolescents in permanent dentition, compared with obese individuals in the same dentition, as classified by the CDC 2000 curve.

## Introduction

Overweight and obesity in children and adolescents used to be considered a condition restricted to high-income countries, but is now increasing worldwide.^1^ Obesity is a multifactorial disease attributed to genetic factors, such as alterations in specific, non-genetic factors, such as lack of physical activity and eating patterns with foods rich in sugars and fats, and to biopsychosocial processes that include political, economic, social and cultural factors.^2,3^ The high prevalence of obesity in the population aged 2 to 18 years has been an important public health problem in both developed and developing countries, since obesity in childhood and adolescence can be perpetuated in adulthood, and may be associated with non-communicable diseases, such as type 2 diabetes, hypertension, and cardiovascular disease.^4,5^

A recent systematic review and meta-analysis investigated the prevalence of overweight and obesity among young Brazilian children and adolescents aged 5 to 19 years. In children (5–9 years), the pooled prevalence rates of overweight were 16.2% in girls and 14.4% in boys. The prevalence rates of obesity were 9.2% and 9.0% for girls and boys, respectively. Regarding adolescents (10–19 years), the prevalence rates in girls were 16.4% for overweight and 6.2% for obesity; in boys, 15.3% for overweight and 7.5% for obesity. The review identified considerable and increasing trends in the prevalence rates of overweight and obesity in the last two decades, with implications for the current health of young people, and for the future health of the entire population, unless something is done to stave off their effects.^6^

Diet is the primary determinant of obesity. A poor diet can have a negative impact on health through its effects on the functioning of the immune system, growth, development, aging, and also on oral health.^7^Among the oral diseases, dental caries is still one of the most highly prevalent chronic diseases among children worldwide.^8,9^In Brazil, according to data from the latest National Survey of Oral Health – SB Brazil 2010, the proportion of individuals without caries experience (dmft/DMF = 0) decreases as age increases, i.e., 46.6% of Brazilian children aged 5 years were free from dental caries in the primary dentition; at age 12, that number dropped to 43.5% for the permanent dentition. From age 15 to 19 years, the number of adolescents free from caries experience was even lower, i.e., 23.9%. It was also found that the percentage of children and adolescents with DMFT = 0 was always lower in the Midwest, North and Northeast, compared with the higher percentage in the South and Southeast.^10^

Dental caries is considered a sugar-dependent polymicrobial dysbiosis, capable of leading to mineral loss of the dental hard tissues.^11^ Dental caries has negative effects on the child’s quality of life, which can lead to discomfort, pain, changes in sleep habits, and poor nutrition. In addition, it can lead to difficulties in the patient’s school performance, socialization and self-esteem, and can compromise the daily life of parents or caregivers.^8,12-15^

Overweight/obesity and dental caries share common risk factors apart from high sugar diet intake, such as lower socioeconomic status (SES), and social-environmental factors, which might explain the association between these two conditions. Moreover, although the scientific literature supports the coexistence of overweight/obesity and dental caries, conflicting results have been described in different populations^7,16^. Several systematic reviews have been undertaken to understand the association of overweight/obesity and dental caries;^17-20^ however, existing evidence remains uncertain and inconclusive.^8^

Hence the present study addresses a focused research question by looking at the evidence for the association between overweight/obesity and dental caries in Brazilian children and adolescents, since a more accurate analysis of this relationship is needed to address the continental dimensions and socioeconomic differences among the different regions of Brazil. This research could support Brazilian health managers in their health decision-making process, in addition to facilitating collaborative and multidisciplinary approaches among specialists involved in the care of children and adolescents. Therefore, the objective of this systematic review and meta-analysis was to evaluate the association between overweight and obesity and dental caries in Brazilian children and adolescents.

## Methodology

### Protocol and registration

A protocol for this study was registered at the International Prospective Register of Systematic Reviews (Prospero) under registration number #CRD42021056843. This systematic review and meta-analysis complies with the Preferred Reporting Items for Systematic Reviews and Meta-Analyses (PRISMA).^21,22^

### Eligibility criteria

The inclusion criteria for this systematic review and meta-analysis were observational studies (cross-sectional, case-control, and cohort studies) and clinical trials that evaluated the association between overweight/obesity and dental caries in Brazilian children and adolescents. The study sample could include children and adolescents ≤ 19 years of age of both sexes, regardless of race, socioeconomic status or region of residence in Brazil. The included studies had to use BMI (Body Mass Index) to assess overweight/obesity. Dental caries experience had to be diagnosed by standardized indices using the visual method, with clinical examinations that evaluated the teeth or surfaces, instead of using radiographic methods.

The PECO question was as follows:

P (Patients): Brazilian children and adolescents ≤ 19 years

E (Exposure): High BMI

C (Comparison): Normal BMI

O (Outcome): Dental caries

Case reports, case series, systematic reviews, abstracts of meetings, or studies whose full texts were unpublished or unavailable were excluded. No restrictions were placed on publication year or publication language.

### Information source and search strategy

Searches were carried out in Cochrane Library, LILACS (Latin American and Caribbean Health Sciences), PubMed, Embase, Scopus, and Web of Science. The searches were conducted as of the date of inception of the database up to July 2023. Keywords and MeSH terms were selected, and electronic search strategies were developed for each database. An additional search in the gray literature (Open Grey) and Google Scholar, and a hand search of the references of the included studies were also performed. The searches in Open Grey and Google Scholar were restricted to the first 300 hits by order of relevance (Table 1).^21^ Endnote software (EndNote X7^®^, Clarivate Analytics, Toronto, Canada) was used to collect references and remove duplicates.

**Table 1:** Search strategies for all the databases.

Pubmed	((Obesity [Mesh] OR overweight [Mesh] OR BMI OR body mass index [Mesh] OR body weight [Mesh]) AND (dental caries [Mesh] OR oral health [Mesh] OR DMF Index [Mesh] OR teeth decay) AND (child [Mesh] OR adolescent [Mesh] OR preschool, child [Mesh]))
Embase	(obesity OR obese OR overweight OR BMI OR “body mass index” OR “body weight”) AND (caries OR “dental caries” OR “DMF Index” OR DMF OR “teeth decay” OR “tooth demineralization”) AND (child OR adolescent OR “child, preschool” OR pediatric)
Web of Science	(obesity OR obese OR overweight OR BMI OR body mass index OR body weight) AND (caries OR dental caries OR DMF Index OR DMF OR teeth decay OR tooth demineralization) AND (child OR adolescent OR child, preschool OR pediatric)
Cochrane	(obesity OR obese OR overweight OR BMI OR body mass index OR body weight) AND (caries OR dental caries OR DMF Index OR DMF OR teeth decay OR tooth demineralization) AND (child OR adolescent OR child, preschool OR pediatric)
Scopus	(obesity OR obese OR overweight OR BMI OR body mass index OR body weight) AND (caries OR dental caries OR DMF Index OR DMF OR teeth decay OR tooth demineralization) AND (child OR adolescent OR child, preschool OR pediatric)
Lilacs	(obesity OR obese OR overweight OR BMI OR body mass index OR body weight) AND (caries OR dental caries OR DMF Index OR DMF OR teeth decay OR tooth demineralization) AND (child OR adolescent OR child, preschool OR pediatric)
SciELO	(obesity OR obese OR overweight OR BMI OR body mass index OR body weight) AND (caries OR dental caries OR DMF Index OR DMF OR teeth decay OR tooth demineralization) AND (child OR adolescent OR child, preschool OR pediatric)
Gray literature	(obesity OR obese OR overweight OR BMI OR body mass index OR body weight) AND (caries OR dental caries OR DMF Index OR DMF OR teeth decay OR tooth demineralization) AND (child OR adolescent OR child, preschool OR pediatric)

### Study selection

The selection of studies was performed by two reviewers (JFP and MVSZ), independently, in two stages. In Stage 1, titles and abstracts were selected according to eligibility criteria using online software (Rayyan, Qatar Computing Research Institute).^23^ Those that appeared to satisfy the eligibility criteria were assessed in Stage 2. The full texts of studies selected in Stage 1 were screened in Stage 2, applying the same criteria. The studies whose full texts fulfilled the eligibility criteria were included. Any discrepancies between the review authors were resolved in both stages with discussion, and a third review author (PVTD) was consulted if discrepancies persisted.

### Data collection process

Data collection was conducted by two independent reviewers (JFP and MVSZ). The following data were extracted: author/year of publication, study design, sample size, participants’ age, aim of the study, the measurements evaluated and the indices used to assess overweight/obesity and dental caries, statistical analyses, results of the association between dental caries and obesity in Brazilian children, and main conclusion of the study. The authors of the included studies were contacted if the required data were incomplete. In cases of incomplete data, the study authors were contacted via the corresponding author’s email address or Research Gate (http://www.researchgate.net/).

### Risk of bias within studies

Risk of bias was assessed with the Joanna Briggs Institute Critical Appraisal Checklist according to the design of the included studies. The Joanna Briggs Institute checklist for analytical cross-sectional studies, the checklist for cohort studies, and the checklist for case-control studies were used.

Two reviewers (JFP and MVSZ) performed the risk of bias evaluation separately, and categorized each article included as a ‘high risk’ study when the study bias rating of ‘low risk of bias’ score was between 0% and 49% of all the items of the tool, a ‘moderate risk’ study when the study bias rating of ‘low risk of bias’ score was between 50% and 69% of all the items of the tool, and a ‘low risk’ study when the study bias rating of ‘low risk of bias’ score was above 70% of all the items of the tool.^24^ In cases of discordance between the two reviewers in rating the bias, a third reviewer (PVTD) was consulted to resolve the disagreement.

### Summary measures

The main outcome assessed was the association between overweight and dental caries, between obesity and dental caries, and between overweight/obesity and dental caries. The summary measure considered the odds ratios (OR) in dichotomous variables, with 95% confidence intervals (CI). Regarding continuous variables, the MD and median range (MR) were considered, as well as 95%CI.

### Synthesis of results

Fifteen meta-analyses were conducted with the Review Manager 5.4 (Review Manager 5.4, The Cochrane Collaboration) software. Statistical heterogeneity was quantified using the I^2^ test, and a value > 50% was considered as an indicator of substantial heterogeneity among studies.^24,25,26^The fixed effect model was used when I^2^ was lower than 50%. The random effect model was used when I^2^ was higher than 50%.^25^The rationale for aggregating studies in different meta-analyses was homogeneity between/among studies according to the dentition (deciduous or permanent) of children/adolescents, the reference curves used to define overweight and obesity in children and adolescents, the classification of the anthropometric variable related to dental caries, and whether the outcome assessed was continuous or dichotomous.

### Strength of the evidence assessment

The strength of evidence of the selected studies for the meta-analyses was assessed using the Grading of Recommendations Assessment, Development and Evaluation (GRADE) system. Summary of Findings (SoF) tables were produced with GRADE online software (GRADEpro GTD, Copenhagen, Denmark).^27^

## Results

### Study selection

**Figure 1:**
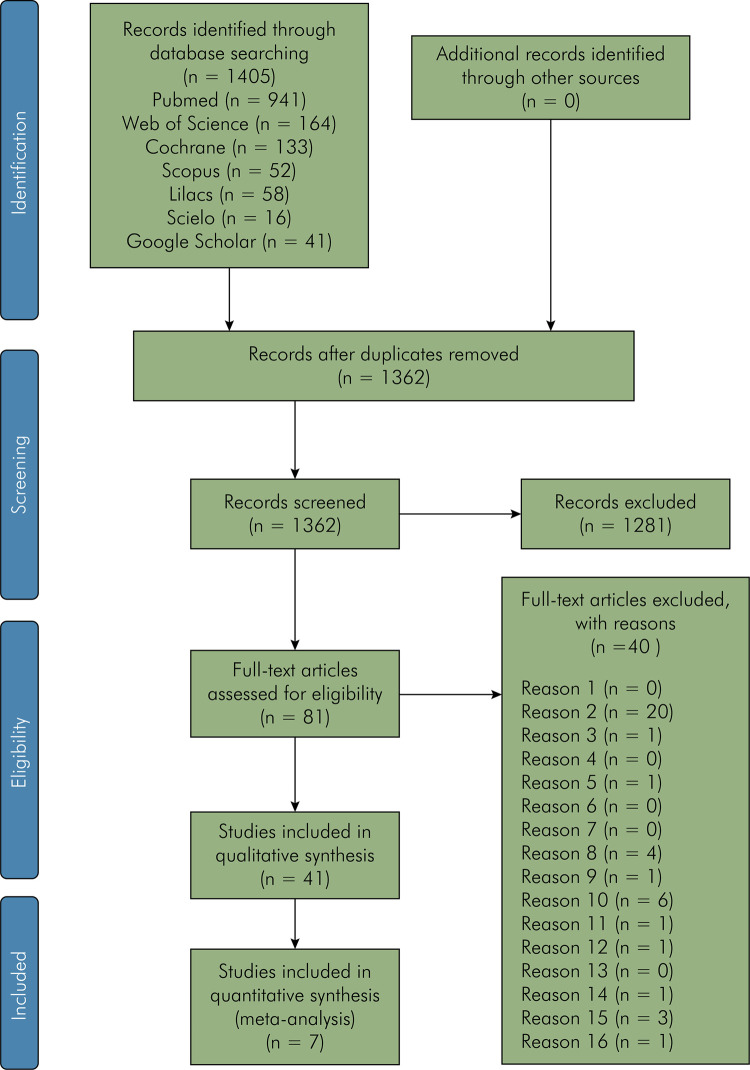
Flow diagram of literature search and selection criteria.

A total of 1,405 references were retrieved within all the searches. After the removal of duplicate hits, 1,362 remained for screening of title and abstract in Stage 1. After screening, 81 references were selected for Stage 2. After applying the eligibility criteria to the full text, 40 articles were excluded. Thus, 41 articles were finally included in this systematic review (Figure 1).

### Study characteristics

Thirty-seven cross-sectional studies, three cohorts, and one case-control study were included. The Brazilian cities and regions where the studies had been performed were: Camboriú,^28^ Londrina,^29,30^ Porto Alegre,^31-33^ Pato Branco,^34^ Pelotas, ^35,36^ Santa Cruz do Sul,^37^ Florianópolis,^38^ Califórnia^39^ and Curitiba^13^ in the South; Diadema,^40^ Juiz de For a,^41^ Piracicaba,^42-44^ Araraquara,^45^ São Paulo,^46,47^ Bauru,^48,49^ Dois Córregos,^50^ Araçatuba,^51^ Nova Friburgo,^52^ Ribeirão Preto,^53^ Diamantina,^54^ and Alfenas^55^ in the Southeast; Goiânia^56^ in the Mid-west; Acrelândia,^57^ Manaus,^58-60^ and Barcelos^61^in the North; Carauru,^62^ Cabo de Santo Agostinho,^63^ Campina Grande,^64,65^ Teresina,^66^ and São Luis^15^ in the Northeast.

The studies were published between 2008 and 2021. The number of participants in the study with the largest sample was 1,528 individuals,^31^ and the number of participants in the study with the smallest sample was 54^38^. The age of the participants ranged from 12 months^33^ to 19 years old.^29,40,66^ Summary information for all the articles can be made available by the authors upon request.

Nutritional status was classified based on BMI in all the studies, and the following reference curves were used to define obesity in children: National Center for Health Statistics,^5,46,62^ Centers for Disease Control and Prevention (NCHS/CDC),^28,29,34,42,53,63^ International Obesity Task Force (IOTF),^41^ WHO/2006,^12,15,30,36,40,46,48,51,54,55,59^ WHO/2007,^31,34,35,38,43,44,47,49,51,53,55-57, 60,61,64,66^ Hammer et al., criteria,^52^ Conde and Monteiro^37^ and Cole et al.^33^

Six studies^28,29,34,42,62,64^ assessed child and adolescent overweight and obesity using the BMI for age and gender percentiles from the Centers for Disease Control and Prevention 2000 (CDC 2000) growth charts for children and adolescents from 2 to 20 years old (overweight: ≥ 85^th^ percentile < 95^th^ percentile, and obesity: ≥ 95^th^ percentile), while one paper^52^ used similar parameters based on Hammer et al. Ten studies used BMI for age and gender according to WHO for children younger than five years old, expressed by percentiles (overweight: ≥ 85^th^percentile < 97^th^ percentile, and obesity: ≥ 97^th^ percentile)^30,48,51,54,55^or by Z-score (overweight: Z-score > 1, and obesity: Z-score > 2).^14-16,40,59^ One study used BMI for age and gender according to WHO, but did not report the classification used (percentile or Z-score).^51^ Seventeen studies used BMI for age and gender according to WHO (2007) for children and adolescents between 5 and 19 years, expressed by percentiles (overweight: ≥ 85^th^percentile < 97^th^ percentile, and obesity: ≥ 97^th^ percentile)^38,47,49,50,53^ or by Z-score (overweight: Z-score > 1, and obesity: Z-score > 2).^31,32,35,56-58,60,61,64,66^ Two studies used BMI for age and gender according to WHO (2007), but did not report the classification used (percentile or Z-score).^43,44^ In four studies, overweight and obesity were identified from the curves equivalent to BMI 25.0 kg/m^2^ and 30.0 kg/m^2^, respectively, as recommended by Cole et al. (2000)^33,41,45^ and by Conde and Monteiro.^37^ One study^65^ assessed child and adolescent overweight using standards for adult overweight (BMI > 24.9 kg/m^2^) recommended by the WHO. In one study, the reference curve that had been used to assess childhood overweight and obesity based on BMI was not reported.^39^

Twenty-nine studies assessed dental caries using the DMFT/dmft indices;^36,12,,28-31,33,35,37-39,41-43,45,48-53,57,58,61,62,64^ four studies used the decayed component of the DMFT/dmft;^50,57,60,63^ one study used the dft (decayed and filled primary teeth),^46^ and one study used the disaggregated components of the dmft index.^58^ Two studies evaluated decayed, missing, or filled surfaces in primary teeth (dmfs).^32,40^ One study assessed early childhood caries (ECC) (including cavitated and active non-cavitated lesions, as well as missing teeth and filled cavities) in the primary teeth of children younger than 71 months of age.^67^ Two studies took into consideration the severity of dental caries in their analyses using dmft ≥ 6 (S-ECC)^52^and dmfs ≥ 6 (S-ECC).^40^Three studies assessed caries severity with the Significant Caries Index (SiC index).^41,50,56^ Five studies assessed dental caries using the ICDAS index.^47,54,55,64,67^ White spot lesions (WSL) were also evaluated in one study.^52^

### Results of individual studies

#### Overweight vs. dental caries

Twenty-three studies tested the association between overweight and dental caries.^12,36,29,30,32,34,35,37,39,43,44,47,48,52,53,55-61,66^ Of these, 21 found no association between the two variables investigated in children/adolescents.^12,36,29,30,32,34,37,39,43-45,48,51-53,56-60,66^ Three studies concluded that overweight children/adolescents were less likely to exhibit dental caries than their normal weight peers.^47,55,61^

#### Obesity vs. dental caries

Twenty-three studies tested the association between obesity and dental caries.^36,29,30,32,34,37,39,40,43-46,48,51-53,55,57-60,63,65^Of these, 18 found no association between these two variables investigated in children/adolescents.^36,29,30,34,37,39,40,43-45,48,51-53,57,58,60,61^Three studies demonstrated that obesity was associated with greater dental caries experiences in children/adolescents.^46,59,63^ In one of these three studies, the measurement of the association was limited to bivariate analysis.^63^ Two studies showed that obesity was associated with fewer dental caries experiences in children/adolescents.^32,55^

#### Overweight/obesity vs. dental caries

Fourteen studies tested the association between overweight/obesity and dental caries.^28,31,35,33,41,42,49,50,54,55,62,64,65,67^Of these, 10 found no association between these two variables investigated in children/adolescents.^28,31,35,41,42,49,55,62,64,65^ Four studies demonstrated that overweight/obesity was associated with fewer dental caries experiences in children/adolescents.^15,33,50,54^ In one of these four studies, the measurement of the association was limited to bivariate analysis.^50^

#### Risk of bias within the studies

In the cross-sectional studies, 30 studies exhibited low risk of bias,^12,15,36,28,29,31,34,33,37,38,40-43,45,47-50,52-58,60,61,53,54^ while five studies exhibited moderate risk of bias^30,39,43,46,59^. In addition, two studies showed a high risk of bias.^51,65^ Eleven studies increased the risk of bias when evaluating the reliability of the exposure measure.^30,35,37,39,43,44,47,51,54,55,65^ Of these, one^30^ did not report the anthropometric curve used for BMI index, and 10^30,35,37,43,44,47,51,54,55,65^ showed errors in the description of parameters to assess obesity and overweight using the BMI index. In general, the question that most commonly contributed to increasing the risk of bias was “Were the strategies to deal with confounding factors stated?” Only 22 studies declared strategies to deal with confounders.^12,31,33,37,38,40,43,45,49,50,53,57,60,61,63,64,66^The same occurred with the case-control^62^ study, which was considered as having a low risk of bias, and which received a negative score only for the question about strategies to deal with stated confounding factors. In the cohort studies, all the studies exhibited low risk of bias,^15,32,35^ and the confounding factors were controlled in all of them. Further information about the criteria for scoring the questions as ‘low risk of bias’ or ‘high risk of bias’ can be found in Table 2, Table 3 and Table 4.

**Table 2:** Risk of bias for cross-sectional studies.

Author, year	Were the criteria for inclusion in the sample clearly defined?	Were the study subjects and the setting described in detail?	Was the exposure measured in a valid and reliable way?	Were objective, standard criteria used to measure the condition?	Were the confounding factors identified?	Were the strategies to deal with confounding factors stated?	Were the outcomes measured in a valid and reliable way?	Was appropriate statistical analysis used?
Oliveira; Sheiham; Bönecker, 2008^40^	Yes	Yes	Yes	Yes	Yes	Yes	Yes	Yes
Carvalho *et al.*, 2009^41^	Yes	Yes	Yes	Yes	No	No	Yes	Yes
Crispim *et al.,* 2010^28^	Yes	Yes	Yes	Yes	Yes	No	Yes	Yes
Tambelini *et al.,* 2010^29^	Yes	Yes	Yes	Yes	Yes	No	Yes	Yes
Tureli, Barbosa; Gavião, 2010^42^	Yes	Yes	Yes	Yes	No	No	Yes	Yes
Silva *et al.,* 2013^16^	Unclear	Yes	Unclear	Unclear	Yes	Yes	Yes	Yes
Campos *et al.*, 2011^45^	Yes	Yes	Yes	Yes	Yes	No	Yes	Yes
Alves *et al.,* 2013^31^	Yes	Yes	Yes	Yes	Yes	Yes	Yes	Yes
Costa; Daher; Queiroz, 2013^56^	Yes	Yes	Yes	Yes	Yes	No	Yes	Yes
Xavier *et al.,* 2013^48^	Yes	Yes	Yes	Yes	Yes	No	Yes	Yes
Santos Junior *et al.*, 2014^63^	Yes	Yes	Yes	Yes	Yes	Yes	Yes	Yes
Frazão *et al.,* 2014^57^	Yes	Yes	Yes	Yes	Yes	Yes	Yes	Yes
Freitas *et al.,* 2014^50^	Yes	Yes	Yes	Yes	Yes	Yes	Yes	Yes
Lima *et al.,* 2014^34^	Yes	Yes	Yes	Yes	Yes	No	Yes	Yes
Martins *et al.,* 2014^51^	Yes	Yes	Yes	Yes	Unclear	No	Yes	Yes
Aznar, 2015^49^	Yes	Yes	Yes	Yes	Yes	Yes	Yes	Yes
Antunes *et al.,* 2016^52^	Yes	Yes	No	No	Yes	No	Yes	Yes
Aragão *et al.*, 2016^65^	Yes	No	Yes	Yes	No	No	Yes	Yes
Assi *et al.*, 2016^58^	Yes	Yes	Yes	Yes	Yes	No	Yes	Yes
Silva *et al.,* 2016^53^	No	No	Yes	Yes	Yes	Yes	Yes	Yes
Borges *et al.*, 2016^37^	Yes	Yes	No	Yes	Yes	Yes	Yes	Yes
Gonçalves *et al.*, 2016^38^	Yes	Yes	Yes	Yes	Yes	Yes	Yes	Yes
Pinto-Sarmento *et al.,* 2016^64^	Yes	Yes	Yes	Yes	Yes	Yes	Yes	Yes
Porcelli *et al.*, 2016^39^	Yes	Yes	Unclear	Yes	Yes	No	Yes	Yes
Araújo *et al.*, 2017^43^	Yes	Yes	Yes	Yes	Yes	Yes	Yes	Yes
Fernández *et al.,* 2017^33^	Yes	Yes	Yes	Yes	Yes	Yes	Yes	Yes
Soares *et al.*, 2017^54^	Yes	Yes	Yes	Yes	Yes	Yes	Yes	Yes
Fraiz *et al.*, 2019^12^	Yes	Yes	Yes	Yes	Yes	Yes	Yes	Yes
Guaré *et al.*, 2019^47^	Yes	Yes	Yes	Yes	Yes	No	Yes	Yes
Lima, 2017^66^	Yes	Yes	Yes	Yes	Unclear	Unclear	Yes	Yes
Porcelli *et al.*, 2019^30^	Yes	Yes	Yes	Yes	Yes	No	Yes	Yes
Vasconcelos *et al.,* 2019^59^	No	Yes	Yes	Yes	No	No	Yes	Yes
Aranha *et al.*, 2020^61^	Yes	Yes	Yes	Yes	Yes	Yes	Yes	Yes
Araujo *et al.,* 2020^44^	Yes	Yes	Yes	Yes	Yes	No	Yes	Yes
Rego *et al.,* 2020^50^	Yes	Yes	Yes	Yes	Yes	Yes	Yes	Yes
Barbosa *et al.*, 2021^55^	Yes	Yes	Yes	Yes	Yes	Yes	Yes	Yes
Shqair *et al.*, 2021^36^	Yes	Yes	Yes	Yes	Yes	No	Yes	Yes

**Table 3:** Risk of bias for case-control studies.

Author, year	Were the groups comparable other than presence of disease in cases or absence of disease in controls?	Were cases and controls matched appropriately?	Were the same criteria used for identification of cases and controls?	Was exposure measured in a standard, valid and reliable way?	Was exposure measured in the same way for cases and controls?	Were the confounding factors identified?	Were the strategies to deal with confounding factors stated?	Were outcomes assessed in a standard, valid and reliable way for cases and controls?	Was the exposure period of interest long enough to be meaningful?	Was appropriate statistical analysis used?
Jamelli; Rodrigues; Lira, 2010^62^	Yes	Yes	Yes	Yes	Yes	Yes	No	Yes	Yes	Yes

**Table 4:** Risk of bias for cohort studies.

Author, year	Were the two groups similar and recruited from the same population?	Were the exposures measured similarly to assign people to both exposed and unexposed groups?	Was the exposure measured in a valid and reliable way?	Were the confounding factors identified?	Were the strategies to deal with confounding factors stated?	Were the groups/participants free of the outcome at the start of the study (or at the moment of exposure)?	Were the outcomes measured in a valid and reliable way?	Was the follow-up time reported and long enough for the outcomes to occur?	Was follow-up complete, and if not, were the reasons to loss to follow-up described and explored?	Were the strategies to address incomplete follow-up used?	Was appropriate statistical analysis used?
Silva, 2014^35^	Not applicable	Not applicable	Yes	Yes	Yes	No	Yes	Yes	Yes	No	Yes
Ribeiro et al., 2017^15^	Not applicable	Not applicable	Yes	Yes	Yes	No	Yes	Yes	Yes	Not applicable	Yes
Lock et al., 2019^32^	Not applicable	Not applicable	Yes	Yes	Yes	No	Yes	Yes	Yes	Yes	Yes

#### Synthesis of results

Meta-analyses were performed according to the reference curves that had been used to define overweight and obesity in children and adolescents.

#### Overweight vs. dental caries

**Figure 2:**
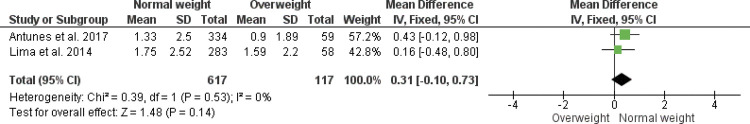
Forest plot of meta-analysis for continuous outcome studies evaluating dental caries in children with primary dentition with normal and overweight using BMI for age and sex percentiles from the 2000 Centers for Disease Control and Prevention (CDC) growth charts.

**Figure 3:**
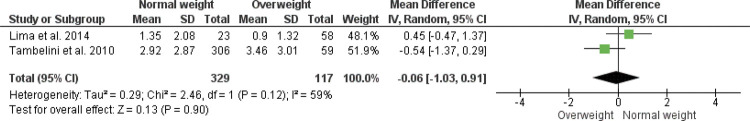
Forest plot of meta-analysis for continuous outcome studies evaluating dental caries in children/adolescents in permanent dentition with normal and overweight using BMI for age and sex percentiles from the 2000 Centers for Disease Control and Prevention (CDC) growth charts.

**Figure 4:**
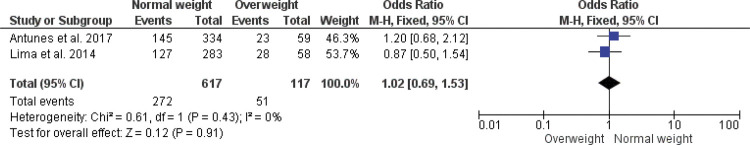
Forest plot of meta-analysis for dichotomous outcome studies evaluating dental caries children in primary dentition with normal and overweight using BMI for age and sex percentiles from the 2000 Centers for Disease Control and Prevention (CDC) growth charts.

**Figure 5:**
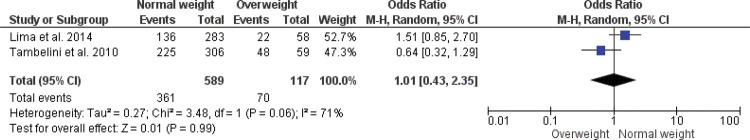
Forest plot of meta-analysis for dichotomous outcome studies evaluating dental caries children/adolescents in permanent dentition with normal and overweight using BMI for age and sex percentiles from the 2000 Centers for Disease Control and Prevention (CDC) growth charts.

Four meta-analyses were performed for the studies that evaluated overweight children and adolescents using BMI for age and sex percentiles from the CDC 2000 growth charts. Two meta-analyses were performed for the continuous outcome studies using the mean difference (MD) and inverse analysis of variance, and two meta-analyses, for dichotomous data using the OR. Regarding continuous outcomes, a meta-analysis with two studies^34,53^ that included children in primary dentition showed that there was no difference between overweight and normal weight children in relation to dental caries (MD = 0.31 95%CI: -0.10 to 0.73; p = 0.14; I^2^= 0%) (Figure 2). Another meta-analysis, which included two studies^29,52^ with children/adolescents in permanent dentition, also showed that there was no difference between overweight and normal weight children/adolescents in relation to dental caries (MD = -0.06 95%CI: -1.03 to 0.91; p = 0.90; I^2^= 59%) (Figure 3). Regarding dichotomous outcomes, a meta-analysis with two studies^41,52^ that included children in primary dentition showed that there was no difference between overweight and normal weight children in relation to dental caries (OR = 1.02 95%CI: 0.69–1.53; p = 0.91; I^2^= 0%) (Figure 4). Another meta-analysis, which included two studies^29,34^with children/adolescents in permanent dentition, also showed that there was no difference between overweight and normal weight children/adolescents in relation to dental caries (OR = 1.01 95%CI: 0.43–2.35;p = 0.99; I^2^= 71%) (Figure 5).

**Figure 6:**
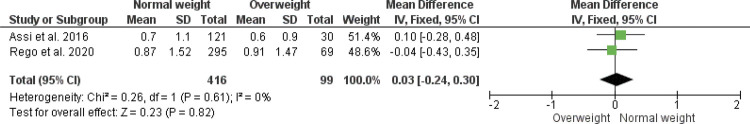
Forest plot of meta-analysis for continuous outcome studies evaluating untreated dental caries in children/adolescents with normal and overweight using BMI for age and sex percentiles from the WHO BMI 2007 expressed by the Z-score.

Of the studies that evaluated overweight using the WHO BMI for age and sex (2007) for children/adolescents between 5 and 19 years old, expressed by the Z-score, a meta-analysis was performed with two studies^58,59^ that evaluated the d component measured as untreated dental caries. No difference between overweight and normal weight children/adolescents was found in relation to untreated dental caries (MD = 0.03 95%CI: -0.24 to 0.30; p = 0.82; I^2^= 0%) (Figure 6).

#### Obesity vs. dental caries

**Figure 7:**
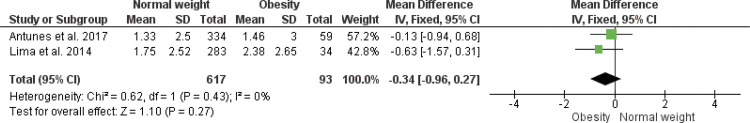
Forest plot of meta-analysis for continuous outcomes studies evaluating dental caries children in primary dentition with normal and obesity using BMI for age and sex percentiles from the 2000 Centers for Disease Control and Prevention (CDC) growth charts.

**Figure 8:**
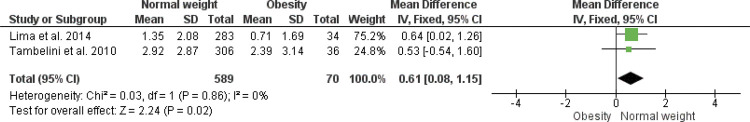
Forest plot of meta-analysis for continuous outcomes studies evaluating dental caries in children/adolescents in permanent dentition with normal and obesity using BMI for age and sex percentiles from the 2000 Centers for Disease Control and Prevention (CDC) growth charts.

**Figure 9:**
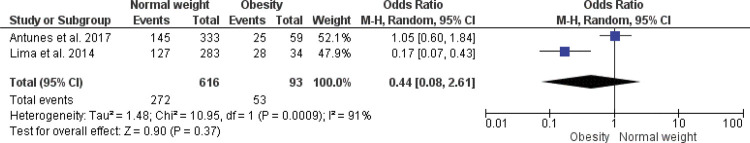
Forest plot of meta-analysis for dichotomous outcome studies evaluating dental caries children in primary dentition with normal and obesity using BMI for age and sex percentiles from the 2000 Centers for Disease Control and Prevention (CDC) growth charts.

**Figure 10:**
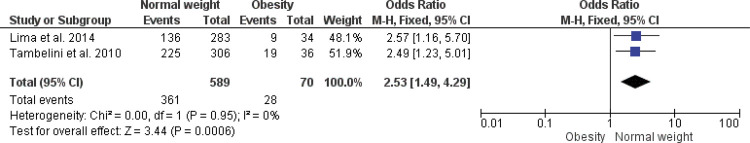
Forest plot of meta-analysis for dichotomous outcome studies evaluating dental caries children/adolescents in permanent dentition with normal and obesity using BMI for age and sex percentiles from the 2000 Centers for Disease Control and Prevention (CDC) growth charts.

Four meta-analyses were performed with studies that evaluated obese children and adolescents using BMI for age and sex percentiles from the CDC 2000 growth charts, two meta-analyses for continuous outcomes studies using the MD and inverse analysis of variance method, and two meta-analyses for dichotomous data using the OR. Regarding continuous outcomes, a meta-analysis with two studies^34,52^ with children in primary dentition showed that there was no difference between obese and normal weight children in relation to dental caries (MD = -0.34 95%CI: -0.96 to 0.27; p = 0.27; I^2^= 0%) (Figure 7). Another meta-analysis comprising two studies^29,34^ with children/adolescents in permanent dentition, showed a higher mean of dental caries in children/adolescents with normal weight (MD = 0.61 95%CI: 0.08–1.15; p = 0.02; I^2^= 0%) (Figure 8). Regarding dichotomous data, a meta-analysis made up of two studies^34,52^ with children in primary dentition showed that there was no difference between obese and normal weight children in relation to dental caries (OR = 0.44 95%CI: 0.08–2.61; p = 0.37; I^2^= 91%) (Figure 9). Another meta-analysis comprising two studies^29,34^ with children/adolescents in permanent dentition, showed a higher odds of dental caries in children/adolescents with normal weight (OR = 2.53 95% CI 1.49-4.29;p = 0.0006; I^2^ = 0%) (Figure 10).

**Figure 11:**
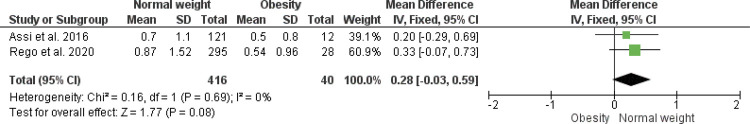
Forest plot of meta-analysis for continuous outcome studies evaluating untreated dental caries in children/adolescents with normal and obesity using BMI for age and sex percentiles from the WHO BMI 2007 expressed by the Z-score.

Of the studies that evaluated obesity using the WHO BMI for age and sex (2007) for children/adolescents, and expressed by the Z-score, a meta-analysis was performed with two studies^58,59^ that evaluated the d component measured as untreated dental caries. No difference was found between overweight and normal weight children/adolescents in relation to untreated dental caries (MD = 0.20 95%CI: -0.03 to 0.59; p = 0.08; I^2^= 0%) (Figure 11).

#### Overweight/obesity vs. dental caries

**Figure 12:**
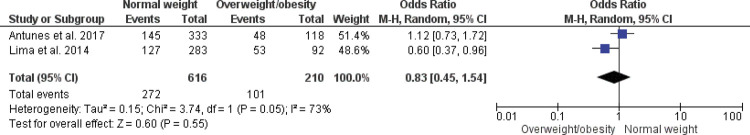
Forest plot of meta-analysis for dicothomous outcomes studies evaluating dental caries children in primary dentition with normal and overweight/obesity using BMI for age and sex percentiles from the 2000 Centers for Disease Control and Prevention (CDC) growth charts.

**Figure 13:**
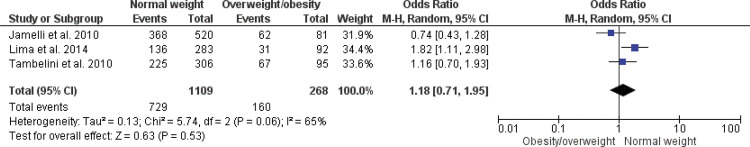
Forest plot of meta-analysis for dichotomous outcomes studies evaluating dental caries children/adolescents in permanent dentition with normal and overweight/obesity using BMI for age and sex percentiles from the 2000 Centers for Disease Control and Prevention (CDC) growth charts.

Of the studies that evaluated overweight/obese children and adolescents using BMI for age and sex percentiles from the CDC 2000 growth charts, two meta-analyses were performed to determine continuous outcome. One meta-analysis with two studies^34,52^ included children in primary dentition. It showed that there was no difference between overweight/obese and normal weight children in relation to dental caries (OR = 0.83 95%CI: 0.45–1.54; p = 0.55; I^2^= 73%) (Figure 12). Another meta-analysis included three studies^29,34,62^ with children/adolescents in permanent dentition, and showed no difference between overweight/obese and normal weight children in relation to dental caries (OR = 1.18 95%CI: 0.71-1.95; p = 0.53; I^2^= 65%) (Figure 13).

**Figure 14:**
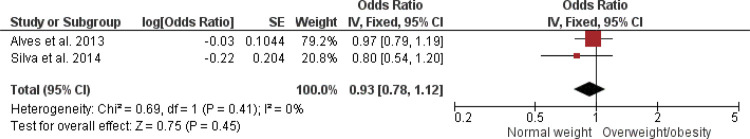
Forest plot of meta-analysis for dichotomous outcome studies evaluating dental caries in children/adolescents with normal and overweight/obesity using BMI for age and sex percentiles from the WHO BMI 2007 expressed by the Z-score.

A meta-analysis with two studies was performed for the studies that evaluated overweight/obesity using the WHO BMI for age and sex (2007) for children/adolescents, expressed by the Z-score.^31,34^ No difference was found between overweight/obese and normal weight children/adolescents in relation to dental caries (OR = 0.93 95%CI: 0.78–1.12; p = 0.45; I^2^= 0%) (Figure 14).

**Figure 15:**
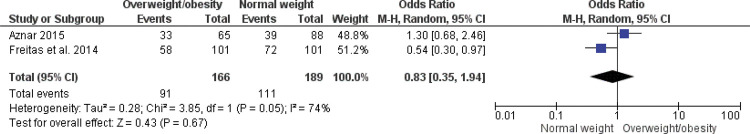
Forest plot of meta-analysis for dichotomous outcome studies evaluating dental caries in children/adolescents with normal and overweight/obesity using BMI for age and sex percentiles from the WHO BMI 2007 expressed by the percentiles.

A meta-analysis with two studies was performed for the studies that evaluated overweight/obesity using the WHO BMI for age and sex (2007) for children/adolescents, expressed by the percentiles.^49,50^ No difference was found between overweight/obese and normal weight children/adolescents in relation to dental caries (OR = 0.83 95%CI: 0.35–1.94; p = 0.67; I^2^= 74%) (Figure 15).

**Figure 16:**
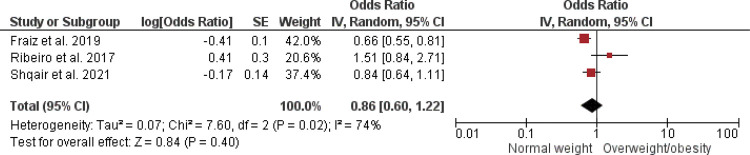
Forest plot of meta-analysis for dichotomous outcome studies evaluating dental caries in children with normal and overweight/obesity using BMI for age and sex percentiles from the WHO BMI 2006 expressed.

A meta-analysis with three studies was performed for the studies that evaluated overweight/obesity using the BMI for age and gender classified by WHO for children younger than five years, expressed by the Z-score.^12,15,36^ No difference was found between overweight/obese and normal weight children/adolescents in relation to dental caries (OR = 0.86 95%CI: 0.60–1.22; p = 0.40; I^2^= 74%) (Figure 16).

#### Strength of the evidence assessment

Based on the GRADE assessment, the strength of evidence was rated as very low for the results of the meta-analyses that used the anthropometric reference curves: CDC 2000 for obesity in children with primary teeth, CDC 2000 for overweight and obesity in children/adolescents with permanent and deciduous teeth, and CDC 2000 for overweight children and adolescents with permanent and deciduous teeth; and those that used the WHO 2007 percentile for overweight and obese children and adolescents with permanent teeth, and the WHO 2006 Z-Score for overweight and obese children with primary and permanent teeth. The strength of evidence was rated as low for the results of the meta-analyses that used the anthropometric reference curve WHO 2007 Z-Score to assess obesity in children with untreated dental caries, the WHO 2007 Z-Score to assess overweight in children with untreated dental caries, and the WHO 2007 Z-Score to assess obese and overweight children and teenagers with permanent teeth. The strength of evidence was rated as moderate for the meta-analysis outcome that used the CDC 2000 anthropometric reference curve for obesity in children/adolescents in permanent dentition. Additional information on the strength of evidence assessment is available in Tables 5, 6, 7 and 8.

**Table 5:** Assessment of certainty of evidence of studies that used the WHO 2007 Z score as an anthropometric curve of the BMI

Certainty assessment							No. of patients		Effect		Certainty	Importance
No. of studies	Study design	Risk of bias	Inconsistency	Indirectness	Imprecision	Other considerations	Obesity/ Overweight and Obesity/ Overweight	Normal Weight	Relative (95% CI)	Absolute (95% CI)		
WHO 2007 Z Score_Obesity_D_Mean												
2	observational studies	serious^a^	not serious	not serious	not serious	all plausible residual confounding would reduce the demonstrated effect	40	416	-	MD 0.28 higher	⨁⨁◯◯	IMPORTANT
										(0.03 lower to 0.59 higher)	Low	
WHO 2007 Z Score_Overweight_D_Mean												
2	observational studies	serious^b^	not serious	not serious	not serious	all plausible residual confounding would reduce the demonstrated effect	99	416	-	MD 0.03 higher	⨁⨁◯◯	IMPORTANT
										(0.24 lower to 0.3 higher)	Low	
WHO 2007 Z Score_Overweight and Obesity_DMFT_Dichotomous												
2	observational studies	not serious	not serious	not serious	not serious	none			not estimable	not estimable	⨁⨁◯◯	IMPORTANT
							-	0.0%			Low	

CI: confidence interval; MD: mean difference.

a. One of the included studies did not mention the strategies used to deal with confounders; b. One of the included studies did not mention the strategies used to deal with confounders.

**Table 6:** Assessment of the certainty of the evidence of studies that used WHO 2007 percentile as an anthropometric curve of BMI

Certainty assessment							No. of patients		Effect		Certainty	Importance
No. of studies	Study design	Risk of bias	Inconsistency	Indirectness	Imprecision	Other considerations	Overweight and Obesity	Normal weight	Relative	Absolute		

									(95% CI)	(95% CI)		
WHO 2007 Percentile_Overweight and Obesity_DMFT_Dichotomous												
2	observational studies	not serious	serious^a^	not serious	serious^b^	none	91/166 (54.8%)	111/189 (58.7%)	OR 0.83	46 fewer per 1,000	⨁◯◯◯	IMPORTANT
									(0.35 to 1.94)	(from 255 fewer to 147 more)	Very low	

CI: confidence interval; OR: odds ratio.

a. i^2^ = 74. There is no effect estimate similarity to overlapping confidence intervals; b. Although the CI is small (0.35–1.94), the number of events is fewer than 300.

**Table 7:** Assessment of certainty of evidence of studies that used the WHO 2006 z score as an anthropometric curve of the BMI.

Certainty assessment							No. of patients		Effect		Certainty	Importance
No. of studies	Study design	Risk of bias	Inconsistency	Indirectness	Imprecision	Other considerations	Obesity/ Overweight and Obesity/ Overweight	Normal Weight	Relative (95% CI)	Absolute (95% CI)		
WHO 2006 Z Score_Overweight and Obesity_dmft_Dichotomous												
3	observational studies	serious^a^	serious^b^	serious^c^	not serious	none			not estimable		⨁◯◯◯	IMPORTANT
											Very low	
WHO 2006 Z Score_Overweight and Obesity_DMFT_DIchotomous												
2	observational studies	not serious	serious^d^	not serious	not serious	none			not estimable		⨁◯◯◯	IMPORTANT
											Very low	

CI: confidence interval.

a. Some included studies did not mention the strategies used to deal with confounders. One study did not report whether groups/participants were outcome-free at baseline (or at the time of exposure); b. i^2^ = 74%. There is no similarity of effect estimates to overlapping confidence intervals; c. Although the results refer only to the primary dentition, the children included in the studies ranged from 2–8 years old; d. i^2^ = 85%. There is no similarity of effect estimates to overlapping confidence intervals.

**Table 8:** Assessment of the certainty of the evidence of studies that used the CDC 2000 score as an anthropometric curve of the BMI.

Certainty assessment							No. of patients		Effect		Certainty	Importance
No. of studies	Study design	Risk of bias	Inconsistency	Indirectness	Imprecision	Other considerations	Obesity/ Overweight and Obesity/ Overweight	Normal Weight	Relative (95%CI)	Absolute (95%CI)		
CDC 2000_Obesity_DMFT_Dichotomous												
2	observational studies	serious^a^	not serious	serious^b^	not serious	very strong association	28/70 (40.0%)	361/589 (61.3%)	OR 2.53	187 more per 1.000	⨁⨁⨁◯	IMPORTANTE
						all plausible residual confounding would reduce the demonstrated effect			(1.49 to 4.29)	(from 89 more to 259 more)	Moderate	
CDC 2000_Obesity_dmft_Dichotomous												
2	observational studies	serious^c^	serious^d^	serious^e^	serious^f^	all plausible residual confounding would reduce the demonstrated effect	53/93 (57.0%)	272/616 (44.2%)	OR 0.44	183 fewer per 1.000	⨁◯◯◯	IMPORTANTE
									(0.08 to 2.61)	(from 382 fewer to 232 more)	Very low	
CDC 2000_Obesity_DMFT_Mean												
2	observational studies	serious^g^	not serious	serious^h^	not serious	all plausible residual confounding would reduce the demonstrated effect	70	589	-	MD 0 0.61	⨁◯◯◯	IMPORTANTE
										(0.08 higher to 1.15 higher)	Very low	
CDC 2000_Obesity_dmft_Mean												
2	observational studies	serious^i^	not serious	serious^j^	not serious	all plausible residual confounding would reduce the demonstrated effect	93	617	-	MD 0 -0.34	⨁◯◯◯	IMPORTANTE
										(0.96 lower to 0.27 higher)	Very low	
CDC 2000_Overweight and Obesity_DMFT_Dichotomous												
3	observational studies	serious^k^	serious^l^	serious^m^	not serious	strong association	160/268 (59.7%)	729/1109 (65.7%)	OR 1.18	36 more per 1.000	⨁◯◯◯	IMPORTANTE
						all plausible residual confounding would reduce the demonstrated effect			(0.71 to 1.95)	(from 81 fewer to 132 more)	Very low	
CDC 2000_Overweight and Obesity_dmft_Dichotomous												
2	observational studies	serious^n^	serious^o^	serious^p^	serious^q^	all plausible residual confounding would reduce the demonstrated effect	101/210 (48.1%)	272/616 (44.2%)	OR 0.83	45 fewer per 1.000	⨁◯◯◯	IMPORTANTE
									(0.45 to 1.54)	(from 179 fewer to 108 more)	Very low	
CDC 2000_Overweight_DMFT_Dichotomous												
2	observational studies	serious^r^	serious^s^	serious^t^	serious^u^	strong association	70/117 (59.8%)	361/589 (61.3%)	OR 1.01	2 more per 1.000	⨁◯◯◯	IMPORTANTE
						all plausible residual confounding would reduce the demonstrated effect			(0.43 to 2.35)	(from 208 fewer to 175 more)	Very low	
CDC 2000_Overweight_dmft_Dichotomous												
2	observational studies	serious^v^	not serious	serious^w^	serious^x^	strong association	51/117 (43.6%)	272/617 (44.1%)	OR 1.02	5 more per 1.000	⨁◯◯◯	IMPORTANTE
						all plausible residual confounding would reduce the demonstrated effect			(0.69 to 1.53)	(from 89 fewer to 106 more)	Very low	
CDC 2000_Overweight_DMFT_Mean												
2	observational studies	serious^y^	serious^z^	serious^aa^	serious^ab^	all plausible residual confounding would reduce the demonstrated effect	117	329	-	MD 0.06 lower	⨁◯◯◯	IMPORTANTE
										(1.03 lower to 0.91 higher)	Very low	
CDC 2000_Overweight_dmft_Mean												
2	observational studies	serious^ac^	not serious	serious^ad^	serious^ae^	all plausible residual confounding would reduce the demonstrated effect	117	617	-	MD 0.31 higher	⨁◯◯◯	IMPORTANTE
										(0.1 lower to 0.73 higher)	Very low	

CI: confidence interval; MD: mean difference; OR: odds ratio.

a. The included studies did not mention the strategies used to deal with confounders. Thus, we believe that the studies have serious methodological limitations; b. Although the results refer only to permanent dentition, the children and adolescents of the included studies ranged from 6–19 years old; c. The included studies did not mena.tion the strategies used to deal with confounders. Thus, we believe that the studies have serious methodological limitations; d. i^2^ = 91%; e. Although the results refer only to the primary dentition, the children included in the studies ranged from 2 –15 years old; f. The number of effects was less than 300. The CI ranged from 0.08–2.61. The prism passes through the null line; g. Although the results refer only to permanent dentition, the children and adolescents of the included studies ranged from 6–19 years old; h. The included studies did not mention the strategies used to deal with confounders. Thus, we believe that the studies have serious methodological limitations; i. The included studies did not mention the strategies used to deal with confounders. Thus, we believe that the studies have serious methodological limitations; j. Although the results refer only to the primary dentition, the children included in the studies ranged from 2–15 years old; k. The included studies did not mention the strategies used to deal with confounders. Thus, we believe that the studies have serious methodological limitations. Two of the studies included in the meta-analysis are cross-sectional and one is a case-control study; l. i^2^ = 65% and there is no similarity of effect estimates on overlapping confidence intervals; m. Although the results refer only to permanent dentition, the children and adolescents of the included studies ranged from 6–19 years old; n. The included studies did not mention the strategies used to deal with confounders. Thus, we believe that the studies have serious methodological limitations; o. i^2^ = 73% and there is no similarity of effect estimates on overlapping confidence intervals; p. Although the results refer only to the primary dentition, the children included in the studies ranged from 2–15 years old; q. Although the CI is small (0.45–1.54), the number of events is less than 300; r. The included studies did not mention the strategies used to deal with confounders. Thus, we believe that the studies have serious methodological limitations; s. i^2^ = 71% and there is no similarity of effect estimates on overlapping confidence intervals; t. Although the results refer only to permanent dentition, the children and adolescents of the included studies ranged from 6–19 years old; u. The number of effects was less than 300. The CI ranged from 0.43–2.35. The prism passes through the null line; v. The included studies did not mention the strategies used to deal with confounders. Thus, we believe that the studies have serious methodological limitation; w. Although the results refer only to the primary dentition, the children included in the studies ranged from 2 to 15 years old; x. Although the CI is small (0.69–1.53), the number of events is less than 300; y. The included studies did not mention the strategies used to deal with confounders. Thus, we believe that the studies have serious methodological limitations; z. i^2^ = 5 9% and there is no similarity of effect estimates on overlapping confidence intervals; aa. Although the results refer only to permanent dentition, the children and adolescents of the included studies ranged from 6–19 years old; ab. Although the sample was greater than 400, the CI was (-1.03 to 0.91). The prism passes through the null line; ac. The included studies did not mention the strategies used to deal with confounders. Thus, we believe that the studies have serious methodological limitations; ad. Although the results refer only to the primary dentition, the children included in the studies ranged from 2–15 years old; ae. Although the sample was greater than 400, the CI was (-0.10 to 0.73). The prism passes through the null line.

## Discussion

The current systematic review and meta-analysis provides information on the association between overweight and obesity (as determined by BMI) and dental caries in Brazilian children and adolescents. The results indicated that the evidence of an association between overweight and obesity and dental caries in Brazilian children and adolescents is contrasting and not consistent, as evaluated in other systematic reviews in which studies from several countries in the world were included.^7,16,67-69^ The data of the meta-analyses showed no association between obesity, overweight, overweight/obesity and dental caries for most anthropometric reference curves used to assess BMI, and had very low or low strength of evidence. Moderate strength of evidence was found for the meta-analysis showing a greater experience of dental caries in the permanent dentition of normal weight adolescents, compared with obese individuals, as classified by the CDC 2000 curve.

Few studies included in this systematic review and meta-analysis showed an association between obesity, overweight, overweight/obesity and greater experience of dental caries in Brazilian children/ adolescents.^46,69^The primary studies that included this association reported an increased prevalence and/or severity of caries in overweight/obese individuals who consumed particularly high levels of carbohydrates.^46,62^This interpretation cognizes sugar as an etiological factor in caries development. ^59,69^Aside from these explanations, a systematic review^69^ evaluating the association between dental caries and obesity in studies developed worldwide highlighted that Swedish researchers found a reduced salivary flow in obese adolescents, compared with their normal weight peers.^70^ The authors suggested that reduced salivary flow intensifies the development of dental caries, thereby placing obese adolescents at an increased risk of caries.^70^

Nonetheless, the explanation for the non-association between high BMI and dental caries, shown by the primary studies that found this result, is backed by the hypothesis that parents/caregivers of overweight/obese children may restrict the supply of high energy content foods in an attempt to control their children’s weight, and consequently also influence the dental caries experience.^12^ In a Brazilian study, children with excess body weight were submitted to greater snack consumption control.^12^ In this study,^12^ excess body weight was a protective factor against dental caries, when analyzed separately. However, when it was controlled to reflect the level of parental restriction of snack consumption, the association between excess body weight and dental caries lost its statistical significance.^12^ Obesity and dental caries in children have multifactor etiology, and their development involves important social-behavioral components. Nevertheless, the volume, frequency and quality of the foods ingested are the most important factors in obesity,^71^ while the frequency and quality of eating practices have a seemingly greater impact on dental caries than the systemic effect of nutrient intake.^72^

In the literature, there have been other attempts to explain the lower prevalence of dental caries in obese individuals. One such endeavor has suggested that the observed association between lower prevalence of dental caries and high BMI may be due to the increased consumption of high-fat and non-high-sugar diets, which are positively associated with obesity rather than dental caries. There are also reports in the literature of a possible protective effect of fatty foods on the frequency of dental caries.^73^ Furthermore, the lower prevalence of dental caries in obese children has been justified by the fact that overweight/obese children have high levels of immunoglobulin A antibody (IgA-s) in their saliva.^20,74^ This is an important factor that influences the microbial adhesion on tooth surfaces, and may hence interfere in the process of caries development and prevention.^32,47,51^

Many studies have also suggested that both caries and obesity are strongly influenced by socioeconomic factors.^28,31,39,49,54,55,57,62,64^Families with better social conditions have greater access to dental treatments, leading to lower dental caries rates. Additionally, these families also have more access to foods that contribute to obesity. According to a national survey exploring data that evaluate nutrient consumption, the caloric participation of lipids in the diet of the population of the Brazilian Southeast is higher than that of other Brazilian regions and the national average.^75^ However, social inequality in Brazil makes it difficult to extrapolate the interpretation of these results to all Brazilians. There are 16 million people who live below the poverty line in Brazil, and who cannot meet their basic food needs, and many regions are affected by food insecurity.^76^ Inappropriate eating habits may interfere with oral health conditions when combined with a lack of inadequate hygiene or difficulties in accessing preventive care. In this regard, inequalities in the oral health of the Brazilian population well portray the very unequal distribution of wealth across the country.^78^ These factors are considered to be potential effect modifiers that can lead to a weak association between obesity and dental caries.

Studies that have evaluated the association between dental caries and obesity indicate that a variety of issues should be discussed before this relationship can be fully understood. The first issue refers to the methods for diagnosing both outcomes.^70^ In an attempt to reduce the heterogeneity between/among studies, the present systematic review and meta-analysis included only studies that evaluated dental caries using the visual method in clinical exams assessing teeth or surfaces, and excluded those that used radiographic methods. There are also aspects that can interfere in the measurement of overweight and obesity. All studies included in this systematic review and meta-analysis evaluated overweight and obesity by means of BMI measurement. There was no study that measured obesity using other diagnostic methods, such as skinfolds, waist circumference, waist-to-hip ratio, or radiographic densitometry (DXA). However, there was great variation between/among the studies in relation to the anthropometric reference curve applied to classify the participant as an overweight or obese individual. These factors may also account for the heterogeneity of the results among the primary studies included herein.

These findings reinforce the evidence that the association between BMI and caries is complex. Differences in methodology, such as experimental design, population and sample size, as well as access to health services, fluoride use, oral health habits, socioeconomic status, diet, dental caries index and BMI classification, should be considered when seeking to explain the conflicting data.^15^

Based on the current published literature, there is no association between obesity and/or overweight and dental caries in Brazilian children/adolescents for most anthropometric reference curves using BMI. A greater experience of dental caries was associated with well-nourished adolescents in permanent dentition compared with obese adolescents, classified by the CDC 2000. Caution should be exercised due to the very low, low, and moderate strength of evidence of the results supporting this association. Assessments with stronger methods and more standardized prospective studies, using a universal measurement system for both overweight/obesity and dental caries, and possible effect-modifying factors are needed to increase the quality of evidence to confirm or negate this possible association, and to help clarify the direction of the association between these two important health conditions.

## Conclusions

It was concluded that there is no association between overweight and/or obesity and dental caries in Brazilian children/adolescents for most anthropometric reference curves using BMI. A greater experience of dental caries was associated with well-nourished adolescents in permanent dentition compared with obese individuals in the same dentition, classified by the CDC 2000 curve for studies. The strength of the evidence of the meta-analysis results was considered very low, low and moderate.
